# Radiative flow of non Newtonian nanofluids within inclined porous enclosures with time fractional derivative

**DOI:** 10.1038/s41598-021-84848-9

**Published:** 2021-03-05

**Authors:** Anas A. M. Arafa, Z. Z. Rashed, Sameh E. Ahmed

**Affiliations:** 1 Department of Mathematics, College of Science and Arts, Qassim University, Al Mithnab, Saudi Arabia; 2 Department of Mathematics, Faculty of Science and Arts, Jouf University, Qurayyat, Saudi Arabia; 3 Department of Mathematics, Faculty of Science, King Khalid University, Abha, Saudi Arabia

**Keywords:** Engineering, Mathematics and computing

## Abstract

An unsteady convection-radiation interaction flow of power-law non-Newtonian nanofluids using the time-fractional derivative is examined. The flow domain is an enclosure that has a free surface located at the top boundaries. Also, the geometry is filled by aluminum foam as a porous medium and the overall thermal conductivity as well as the heat capacity are approximated using a linear combination of the properties of the fluid and porous phases. Additionally, the dynamic viscosity and thermal conductivity of the mixture are expressed as a function of velocity gradients with a fractional power. Marangoni influences are imposed to the top free surface while the bottom boundaries are partially heated. Steps of the solution methodology are consisting of approximation of the time fractional derivatives using the conformable definition, using the finite differences method to discretize the governing system and implementation the resulting algebraic system. The main outcomes reveled that as the fractional order approaches to one, the maximum values of the stream function, the bulk-averaged temperature and cup-mixing temperature are reduces, regardless values of the time.

## Introduction

Svey of the heat transport using the non-Newtonian fluid within domains filled by porous elements is a major topic in the CFD (computational fluid dynamic) filed due to the important practical applications in the real life. These enforcements include oil recovery, material processing, polymer, synthetic lubricants, liquid films, cosmetics, oil–water emulsion, paints, jellies, etc.^1–3^. The power-law non-Newtonian nanofluids are new category of the nanomixtures which contains a non-Newtonain base fluid (For example Carboxymethyl Cellulose (CMC) solutions) and one or more type of the nanoparticles. The purpose of these compounds is to support the thermal transfer of conventional fluids. An enormous number of studies have been presented using Newtonian nanofluids^4–9^ while the interest in the non-Newtonian nanofluids is still weak. The FVM (finite volume method) was applied by Zhuang and Zhu^10^ to study the double diffusive within the cubic enclosure filled with a heterogeneous porous medium. The Marangoni effects together with the CMC-Cuo nanofluids were considered. The findings revealed that the decrease of the power-law index causes an increase in the heat and mass transfer rate as well as intensification in the fluid motion. The heat transfer due to the Marangoni convection using the power –law fluid over a surface saturated porous media has been presented by Jiao et al.^11^. The surface tension is considered as a quadratic function of the gradients of the temperature. It is noticeable that the velocity is reduced while the temperature is increased as the porosity parameter is grown. Lin et al.^12^ investigated the radiation- Marangoni interaction flow using the pseudo-plastic nanofluids having a variable thermal conductivity. The results indicating that the shear stress of CMC-TiO_2_ nanofluid is diminished as the power-law index is altered.

Over the years ago, fractional calculus theory is generalizing the integer order of differentiation to non-integer order. It has many advantageous in several real life fields such as fluid mechanics, optics, plasma, electromagnetism physics, engineering, biology and economics because it shows the new properties of these problems^13–15^. Non- Newtonian fluids dynamics are one of these fields that was modeled by fractional derivatives models because the non-locality of fractional calculus which gives long-term memory^16–25^. However, it is very difficult to achieve the exact solution for nonlinear fractional problems. It is possible to use some numerical and approximate methods to find numerical solutions for most nonlinear fractional problems^26–30^. The natural convection along a vertical wall and cylinder using Caputo time-fractional derivatives are presented in^31,32^. Also, several concepts of fractional derivatives such as fractional logistic models, fractional-Legendre spectral Galerkin method for fractional Sturm–Liouville problems, simulating of COVID-19 using the fractional derivatives and natural convection flow of a fluid using Atangana and Baleanu fractional model are presented in^33–37^. There are many definitions for fractional derivatives and fractional integrals are defined in different ways such as Riemann Liouville, Caputo and others^13–15^. Khalil et al.^38^ presented a new operator called “conformable derivative” which satisfied new conventional properties. Several authors effectively used the conformable operators of fractional order in modelling several models^39–47^. Some recent works use the conformable fractional operator to discuss fractional Newtonian mechanics^26,48^.Tabulated in^48^, the conformable fractional operator $D_{\tau }^{\beta }$ of a function $w\left( {x,y,\tau } \right)$ is denoted as:

$$D_{t}^{\beta } w\left( {x,y,\tau } \right) = \mathop {\lim }\limits_{\theta \to 0} \frac{{w\left( {x,y,\theta \tau^{1 - \beta } } \right) - w\left( {x,y,\tau } \right)}}{\theta },\quad 0\left\langle {\beta \le 1, t} \right\rangle 0.$$

This fractional derivative has the following properties as it is stated in^38–47^:

$$\left( i \right) D_{\tau }^{\beta } \left( c \right) = 0, c\;{\text{ is}}\;{\text{a}}\;{\text{constant}},$$

$$\left( {ii} \right) D_{\tau }^{\beta } \left( {\tau^{k} } \right) = k\tau^{k - \beta } , k \in R,$$

$$\left( {iii} \right) D_{\tau }^{\beta } \left( {aw_{1} + bw_{2} } \right) = a D_{\tau }^{\beta } w_{1} + b D_{\tau }^{\beta } w_{2} ,$$

$$\left( {iv} \right) D_{\tau }^{\beta } \left( {w_{1} \cdot w_{2} } \right) = w_{1} D_{\tau }^{\beta } w_{2} + w_{2} D_{\tau }^{\beta } w_{1} ,$$

$$\left( v \right) D_{\tau }^{\beta } \left( {w_{1} /w_{2} } \right) = \frac{{w_{2} D_{\tau }^{\beta } w_{1} - w_{1} D_{\tau }^{\beta } w_{2} }}{{w_{2}^{2} }},$$

$\left( {vi} \right)If w_{1}$ is differentiable with respect to $\tau$, then $D_{\tau }^{\beta } w_{1} = \tau^{1 - \beta } \frac{{\partial w_{1} }}{\partial \tau },$

where $$w_{1} \left( {x,y,\tau } \right), {\text{and}} w_{2} \left( {x,y,\tau } \right)$$ are $$\beta - {\text{differentiable function at}} \left( {x,y} \right) \in R \times \left( {0,\infty } \right)$$.

In addition to the previous survey, the fractional derivatives are used to simulate the convective flow in various published works^49–59^. Further, the Marangoni convection, non-Newtonian second grade nanofluid flow and non-Newtonian ferrofluid flow are presented in the valuable investigations^60–64^.

This paper aims to use the fractional derivative approaches to examine the radiation and Marangoni influences on the power-law non-Newtonian nanofluid flow within an inclined domains. The geometry has a free surface where the surface tension is a function of the temperature gradients and is filled by a porous medium. The worked liquid is consisting of carboxymethyl cellulose (CMC) as a non-Newtonian base fluid while CuO elements are considered as nanoparticles. Also, the aluminum foam is considered as porous elements while the radiation flux is considered in the normal direction. The conformable operator is used for estimating the time fractional derivatives while the dimensionless governing system is solved numerically using an implicit FDM. The novelty of this work appears in simulating important impacts such as Marangoni influences on the flow of unused nanofluids frequently using the fractional partial differential equations that is did not presented before, and is more attractive for the researchers. Also, the results of the current simulations can be effective in various industrial practices such as oil recovery and materials processing. Further, a good survey on applications of the fractional calculus to oil industry is presented in Martínez-Salgado et al.^65^.

## Description and formulation of the problem

**Figure 1:**
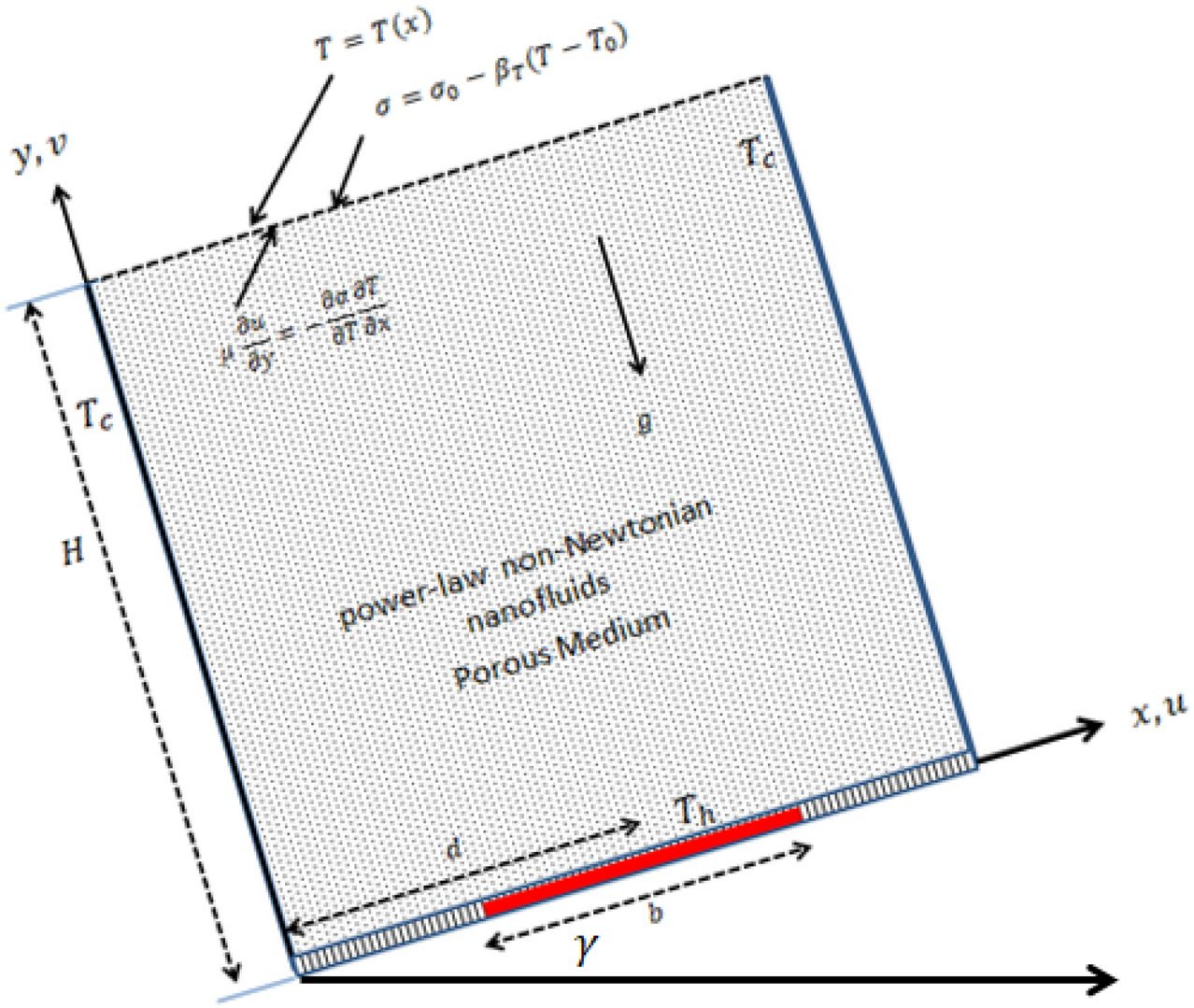
Physical model of the problem.

The flow domain is illustrated in Fig. 1. This situation is consisting of an enclosure that has a free-surface and partially heated from below. The following hypotheses are considered to formulate the mathematical model of this physical case:

- Height of the enclosure is H and the inclination angle is $\gamma$.
- Length of the heated section is $b$ and its location is denoted by $d$.
- A low temperature condition ($$T = T_{c} )$$ is decreed to the side walls and the bottom wall is partially heated ($$T = T_{h}$$) and thermally insulated.
- The free surface (top wall) has a heat transfer based on the Newton's low cooling.
- The surface tension $\sigma$ at the free surface is a function in the nanofluid temperature and it is expressed as:
  1 $$\sigma = \sigma_{0} (1 - \gamma \left( {T - T_{0} } \right)$$

where $$\gamma = - \frac{1}{{\sigma_{0} }}\frac{\partial \sigma }{{\partial T}}$$, $$T_{0} = \frac{{T_{h} + T_{c} }}{2}$$.

- The nanofluid flow is unsteady, laminar and two dimensional.
- The non-Newtonian power law nanofluids are represented by the single-phase model.
- The base fluid is carboxymethyl cellulose (CMC) while CuO is assumed as nanoparticles.
- The thermophysical properties of the components of the nanofluid are given in Table 1 while the dynamical properties of the CMC-water are included in Table 2.
- The domain is filled by homogeneous aluminum foam and the Darcy model is applied.
- The thermal conductivity of the porous medium is considered variable and the thermal radiation is taken in $Y$-direction.
- The thermal equilibrium state is satisfied between the porous and nanofluid phase.

**Table 1:** Properties of the base fluid, porous medium and nanoparticles; see Shah et al.^54^.

Physical properties	CMC	CuO	Aluminum foam
$c_{p}$ (J/kg K)	4179	535.6	897
$\rho$ (kg/m^3^)	997.1	6500	2700
$k$ (W/mK)	0.613	20	205

**Table 2:** Values of dynamical viscosity of the base fluid (CMC-Water), see Zhuang and Zhu^10^.

CMC (%)	n	K (Ns^n^/m^2^)
0.0	1.0	0.000855
0.1	0.91	0.006319
0.2	0.85	0.017540
0.3	0.81	0.0313603
0.4	0.76	0.0785254

The mathematical formulations of the present case are modeled using the continuity, momentum and energy equations based on the previous assumptions; those are written as, see^10,11,66,67^:

2 $$\nabla \cdot {\varvec{V}} = 0$$

3 $$\rho_{nf} \left[ {\frac{1}{\varepsilon }D_{t}^{\beta } {\varvec{V}} + \frac{1}{{\varepsilon^{2} }}{\varvec{V}} \cdot \nabla {\varvec{V}}} \right] = - \nabla p + \nabla \cdot \tau - \frac{{\mu_{eff} }}{K}{\varvec{V}} + \left( {\rho \beta } \right)_{nf} \left( {T - T_{c} } \right){\varvec{g}}$$

4 $$\left[ {\varepsilon \left( {\rho C} \right)_{nf} + \left( {1 - \varepsilon } \right)\left( {\rho C} \right)_{s} } \right]D_{t}^{\beta } T + \left( {\rho C} \right)_{nf} {\varvec{V}} \cdot \nabla T = \nabla \cdot \left( {k_{eff} \nabla T} \right) - \frac{{\partial q_{r} }}{\partial y}$$

5 $$\tau_{ij} = 2\mu_{eff} D_{ij} = \frac{{\mu_{eff} }}{\varepsilon }\left[ {\frac{{\partial V_{i} }}{{\partial x_{j} }} + \frac{{\partial V_{j} }}{{\partial x_{i} }}} \right]$$

6 $$\mu_{eff} = \mu_{nf} .I_{2}^{{0.5\left( {n - 1} \right)}}$$

In Eq. (6), $I_{2} = \frac{1}{2}tr\left( {D^{2} } \right)$ is the second invariant of the deformation tensor where $D = \frac{1}{2}\left[ {\nabla V + \left( {\nabla V} \right)^{T} } \right]$ and $tr$ denotes the trace of a second-order tensor. Here, it should be mentioned that the form of the dynamic viscosity (Eq. 6) is given in Zhuang and Zhu^10^.

Also, $n$ is the power-law index where n < 1 and n > 1 correspond to the case of shear thinning fluids and shear thickening fluids, respectively. More specific:

7 $$I_{2} = \frac{1}{{\varepsilon^{2} }}\left[ {2\left( {\frac{\partial u}{{\partial x}}} \right)^{2} + 2\left( {\frac{\partial v}{{\partial y}}} \right)^{2} + \left( {\frac{\partial u}{{\partial y}} + \frac{\partial v}{{\partial x}}} \right)^{2} } \right]$$

The overall thermal conductivity is depending on the features of the power-law (see Ming et al.^42^) as:

8 $$k_{eff} = \varepsilon \overline{k} + \left( {1 - \varepsilon } \right)k_{s} = \varepsilon \overline{k} + \left( {1 - \varepsilon } \right)k_{s}$$

9 $$\overline{k} = k_{nf} I_{2}^{{0.5\left( {n - 1} \right)}}$$

Introducing the following boundary conditions:

10a $$x = 0, u = 0, v = 0, T = T_{c}$$

10b $$x = H, u = 0, v = 0, T = T_{c}$$

10c $$y = 0 \;{\text{on}}\;{\text{the}}\;{\text{heated}}\;{\text{part}},\quad u = v = 0, T = T_{h}$$

10d $$y = 0\;{\text{on}}\;{\text{the}}\;{\text{insulated}}\;{\text{parts}},\quad u = v = 0, \frac{\partial T}{{\partial y}} = 0$$

10e $$y = H, \overline{\mu }\frac{\partial u}{{\partial y}} = - \frac{\partial \sigma }{{\partial T}}\frac{\partial T}{{\partial x}},v = 0, \frac{\partial T}{{\partial y}} = 0$$

Introducing the next dimensionless quantities:

11 $$\begin{aligned} & X = \frac{x}{H},\quad Y = \frac{y}{H}, \quad U = \frac{u}{{\frac{{\alpha_{f} }}{H}}},\quad V = \frac{v}{{\frac{{\alpha_{f} }}{H}}},\quad \tau = \frac{t}{{\frac{{\alpha_{f} }}{{H^{2} }}}},\quad \theta = \frac{{T - T_{c} }}{{T_{h} - T_{c} }}, \\ & P = \frac{{pL^{2} }}{{\rho_{f} \alpha_{f}^{2} }}, \quad {\text{Da}} = \frac{{k_{f} }}{{H^{2} }},\quad \theta = \frac{{T - T_{c} }}{\Delta T}, \quad {\text{Ra}} = \frac{{g\beta_{f} \left( {T_{h} - T_{c} } \right)H^{3} }}{{\nu_{f}^{ } \alpha_{f} }}, \quad {\text{Pr}} = \frac{{\nu_{f} }}{{\alpha_{f} }}, \quad {\text{Rd}} = \frac{{4 T_{c}^{3} }}{{k_{bf} }}\frac{{\sigma^{*} }}{{ k^{*} }} \\ \end{aligned}$$

The next system is obtained by using Eq. (11)

12 $$\frac{\partial U}{{\partial X}} + \frac{\partial V}{{\partial Y}} = 0$$

13 $$\frac{1}{\varepsilon }D_{\tau }^{\beta } U + \frac{U}{{\varepsilon^{2} }}\frac{\partial U}{{\partial X}} + \frac{V}{{\varepsilon^{2} }}\frac{\partial U}{{\partial Y}} = - \frac{\partial P}{{\partial X}} + \frac{{\rho_{f} }}{{\rho_{nf} }}\frac{{\mu_{nf} }}{{\mu_{f} }}\left[ {\frac{{{\text{Pr}}}}{\varepsilon }\left[ {\frac{{\partial \tau_{XX} }}{\partial X} + \frac{{\partial \tau_{XY} }}{\partial Y}} \right] - \frac{{{\text{Pr}} \mu }}{ Da}U} \right] + {\text{Ra}}\;{\text{Pr}}\frac{{\left( {\rho \beta } \right)_{nf} }}{{\left( {\rho \beta } \right)_{f} }}\frac{{\rho_{f} }}{{\rho_{nf} }}\theta \cos \gamma$$

14 $$\frac{1}{\varepsilon }D_{\tau }^{\beta } V + \frac{U}{{\varepsilon^{2} }}\frac{\partial V}{{\partial X}} + \frac{V}{{\varepsilon^{2} }}\frac{\partial V}{{\partial Y}} = - \frac{\partial P}{{\partial Y}} + \frac{{\rho_{f} }}{{\rho_{nf} }}\frac{{\mu_{nf} }}{{\mu_{f} }}\left[ {\frac{{{\text{Pr}}}}{\varepsilon }\left[ {\frac{{\partial \tau_{XY} }}{\partial X} + \frac{{\partial \tau_{YY} }}{\partial Y}} \right] - \frac{{{\text{Pr}} \mu }}{ Da}V} \right] + {\text{Ra}}\;{\text{Pr}}\frac{{\left( {\rho \beta } \right)_{nf} }}{{\left( {\rho \beta } \right)_{f} }}\frac{{\rho_{f} }}{{\rho_{nf} }}\theta \sin \gamma$$

15 $$\frac{{\left( {1 - \varepsilon } \right)\left( {\rho c_{p} } \right)_{p} + \varepsilon \left( {\rho c_{p} } \right)_{nf} }}{{\left( {\rho c_{p} } \right)_{nf} }}D_{\tau }^{\beta } \theta + U\frac{\partial \theta }{{\partial X}} + V\frac{\partial \theta }{{\partial Y}} = \frac{{\left( {\rho C} \right)_{f} }}{{\left( {\rho C} \right)_{nf} }}\left[ {\frac{\partial }{\partial X}\left( {\frac{{k_{eff} }}{{k_{f} }}\frac{\partial \theta }{{\partial X}}} \right) + \left( {1 + 4\frac{Rd}{3}} \right)\frac{\partial }{\partial Y}\left( {\frac{{k_{eff} }}{{k_{f} }}\frac{\partial \theta }{{\partial Y}}} \right)} \right]$$

16 $$\tau_{xx} = \frac{{2\mu^{ } }}{\varepsilon }\frac{\partial U}{{\partial X}}$$

17 $$\tau_{yy} = \frac{{2\mu^{ } }}{\varepsilon }\frac{\partial V}{{\partial Y}}$$

18 $$\tau_{xy} = \tau_{yx} = \frac{{\mu^{ } }}{\varepsilon }\left[ {\frac{\partial U}{{\partial Y}} + \frac{\partial V}{{\partial X}}} \right]$$

19 $$\mu^{ } = \left[ {2\left[ {\frac{1}{\varepsilon }\frac{\partial U}{{\partial X}}} \right]^{2} + 2\left[ {\frac{1}{\varepsilon }\frac{\partial V}{{\partial Y}}} \right]^{2} + \frac{1}{{\varepsilon^{2} }}\left[ {\frac{\partial U}{{\partial Y}} + \frac{\partial V}{{\partial X}}} \right]^{2} } \right]^{{\frac{n - 1}{2}}}$$

20 $$\frac{{k_{eff} }}{{k_{f} }} = \varepsilon \frac{{\overline{k}}}{{k_{f} }} + \left( {1 - \varepsilon } \right)\frac{{k_{s} }}{{k_{f} }}$$

The dimensionless boundary conditions are:

21a $$X = 0, U = 0, V = 0, \theta = 0$$

21b $$X = 1, U = 0, V = 0, \theta = 0$$

21c $$Y = 0\; {\text{on the heated part}},\quad U = V = 0, \theta = 1$$

21d $$Y = 0 \;{\text{on}}\;{\text{the}}\;{\text{insulated}}\; {\text{parts}},\quad U = V = 0, \frac{\partial \theta }{{\partial Y}} = 0$$

21e $$y = H, \mu \frac{\partial U}{{\partial Y}} = {\text{Ma}}\frac{\partial \theta }{{\partial X}},V = 0, \frac{\partial \theta }{{\partial Y}} = 0$$

In Eq. (21e), ${\text{Ma}} = - \frac{{H\Delta T\frac{\partial \sigma }{{\partial T}}}}{{\mu_{f} \alpha_{f} }}$ is the Marangoni number. Here it should be mentioned that the Roseland approximation is applied for the radiation flux, as follows:

22 $$q_{y} = - \frac{{4\sigma^{*} }}{{3k^{*} }}\frac{{\partial T^{4} }}{\partial y}, \quad T^{4} \cong 4T_{c}^{3} T - 3T_{c}^{4}$$

In Eq. (22), $\sigma^{*}$ is the Stephan–Boltzman constant and $k^{*}$ is the mean absorption coefficient. Also, the following correlations are applied for the nanofluid properties:

23 $$\begin{aligned} & \frac{{\rho_{nf} }}{{\rho_{f} }} = 1 + \phi \left( {\frac{{\rho_{p} }}{{\rho_{f} }} - 1} \right),\quad \frac{{\left( {\rho c_{p} } \right)_{nf} }}{{\left( {\rho c_{p} } \right)_{f} }} = 1 + \phi \left( {\frac{{\left( {\rho c_{p} } \right)_{p} }}{{\left( {\rho c_{p} } \right)_{f} }} - 1} \right),\quad \frac{{\left( {\rho \beta } \right)_{nf} }}{{\left( {\rho \beta } \right)_{f} }} = 1 + \phi \left( {\frac{{\left( {\rho \beta } \right)_{p} }}{{\left( {\rho \beta } \right)_{f} }} - 1} \right) \\ & \frac{{k_{nf} }}{{k_{f} }} = \frac{{\left( {k_{p} + 2k_{f} } \right) - 2\phi \left( {k_{f} - k_{p} } \right)}}{{\left( {k_{p} + 2k_{f} } \right) + \phi \left( {k_{f} - k_{p} } \right)}},\quad \frac{{\mu_{nf} }}{{\mu_{f} }} = \frac{1}{{\left( {1 - \varphi } \right)^{2.5} }} \\ \end{aligned}$$

### Heat transfer coefficients

In the current case, the definition of the local Nusselt number is depending on two sources of the heat flux, namely, the heat flux due to the heated section and the heat flux due to the thermal radiation. The overall heat flux is expressed as:

24 $$q_{y}^{{{\prime \prime \prime }}} = - k_{{eff}} \left. {\frac{{\partial T}}{{\partial y}}} \right|_{{y = 0}} - \frac{{4\sigma ^{*} }}{{3k^{*} }}\left. {\frac{{\partial T^{4} }}{{\partial y}}} \right|_{{y = 0}}$$

Consequently, the local Nusselt number at the heated section is denoted as:

25 $${\text{Nu}} = - \frac{{k_{eff} }}{{k_{f} }}\left( {1 + 4\frac{Rd}{3}} \right)\left. {\frac{\partial \theta }{{\partial Y}}} \right|_{Y = 0}$$

The average Nusselt number for the CMC-nanofluid is defined as follow:

26 $${\text{Nu}}_{av} = - \frac{1}{B}\mathop \int \limits_{D - 0.5B}^{D + 0.5B} {\text{Nu}} dX$$

### Thermal mixing

In this part, the cup-mixing and bulk-averaged temperatures are defined as:

27 $$\theta_{cup} = \frac{{\iint {\overset{\lower0.5em\hbox{$\smash{\scriptscriptstyle\smile}$}}{V} \frac{\delta y}{{\delta x}}\left( {X,Y} \right)\theta_{f} \left( {X,Y} \right)dXdy}}}{{\iint {\overset{\lower0.5em\hbox{$\smash{\scriptscriptstyle\smile}$}}{V} \left( {X,Y} \right)dXdy}}}\quad {\text{where}}\quad \overset{\lower0.5em\hbox{$\smash{\scriptscriptstyle\smile}$}}{V} \left( {X,Y} \right) = \sqrt {U^{2} + V^{2} }$$

28 $$\theta_{avr} = \frac{{\iint {\theta_{f} \left( {X,Y} \right)dXdy}}}{{\iint {dXdy}}}$$

If the non-dimensional temperature changes between 0 and 1, then the value of $\theta_{CUP}$ and $\theta_{avr}$ cannot run over 1.

### Entropy generation

The entropy equations can be writing in the following form:

29 $$s_{gen}^{{{\prime \prime \prime }}} = - \frac{1}{{T_{0}^{2} }}q \cdot \nabla T + \frac{{\mu_{eff} }}{{T_{0} K}}\user2{ }\left( {{\varvec{V}} \cdot {\varvec{V}}} \right) + \frac{{\mu_{eff} }}{{T_{0} }}\left( {\tau_{ij} :\nabla {\varvec{V}}} \right)$$

Using the Fourier law of the heat conduction $\left( {q = - k_{eff} \nabla T} \right)$ and substituting Eq. (24) for the heat flux in Y-direction as well as using the dimensionless variables and the characteristics entropy $\left( {S_{0}^{{{\prime \prime \prime }}} = \frac{{k_{0} \left( {\Delta T} \right)^{2} }}{{H^{2} T_{0}^{2} }}} \right)$, the entropy generation is given by:

30 $$\begin{aligned} & S_{gen}^{{{\prime \prime \prime }}} = \frac{{k_{eff} }}{{k_{f} }}\left[ {\left( {\frac{\partial \theta }{{\partial X}}} \right)^{2} + \left( {1. + 4.\frac{Rd}{3}} \right)\left( {\frac{\partial \theta }{{\partial Y}}} \right)^{2} } \right] + \frac{{\mu_{nf} }}{{\mu_{f} }}\frac{\mu }{Da}{\Theta }\left( {U^{2} + V^{2} } \right) \\ & + \frac{{\mu_{nf } \mu }}{{\mu_{f} }}{\Theta }\left[ {2\left[ {\left( {\frac{\partial U}{{\partial X}}} \right)^{2} + \left( {\frac{\partial V}{{\partial Y}}} \right)^{2} } \right] + \left[ {\frac{\partial U}{{\partial Y}} + \frac{\partial V}{{\partial X}}} \right]^{2} } \right] = S_{T} + S_{F} \\ \end{aligned}$$

In the above equation ${\Theta } = \frac{{\mu_{f} T_{0} }}{{k_{0} }}\left( {\frac{{\alpha_{f} }}{H\Delta T}} \right)^{2}$ is ratio of the irreversibility distribution. In addition the local and average Bejan number are expressed as:

31 $${\text{Be}}\left( {X,Y} \right) = \frac{{S_{T} }}{{S_{{gen}}^{{{\prime \prime \prime }}} }}$$

32 $${\text{Be}}_{av} = \frac{{\mathop \int \nolimits_{A}^{ } Be\left( {X,Y} \right) dA}}{{\mathop \int \nolimits_{A}^{ } dA }}$$

## Numerical method and validation

An implicit scheme based on the finite differences technique is presented for the governing system of the fractional PDE's. Firstly, the time-fractional derivatives are approximated using the conformable definition then the first upwind and the second differences approaches are used for the both the first and second derivatives. The FDM for the time fractional derivatives is expressed as:

33 $${}_{ }^{c} D_{\tau }^{\beta } {\Omega }\left( {x_{i} ,y_{i} ,\tau_{n + 1} } \right) = {\uptau }^{1 - \beta } \frac{{{\Omega }\left( {x_{i} ,y_{i} ,\tau_{n + 1} } \right) - {\Omega }\left( {x_{i} ,y_{i} ,\tau_{n} } \right)}}{{\left( {\Delta {\uptau }} \right)^{ } }}$$

In addition the FDM for the diffusion terms in the RHS of Eqs. (12)-(20) are given as:

34 $$\frac{\partial }{\partial X}\left( {\frac{{\partial {\Omega }}}{\partial X}} \right) + \frac{\partial }{\partial Y}\left( {\frac{{\partial {\Omega }}}{\partial Y}} \right) = \frac{{\left( {\Omega } \right)_{i,j - 1}^{n + 1} - 2\left( {\Omega } \right)_{i,j}^{n + 1} + \left( {\Omega } \right)_{i,j + 1}^{n + 1} }}{{(\Delta Y)^{2} }} + \frac{{\left( {\Omega } \right)_{i + 1,j}^{n + 1} - 2\left( {\Omega } \right)_{i,j}^{n + 1} + \left( {\Omega } \right)_{i - 1,j}^{n + 1} }}{{(\Delta X)^{2} }}$$

Finally, the following algebraic system is obtained:

35 $$A_{p} {\Omega }_{i,j}^{n + 1} = A_{E} {\Omega }_{i + 1,j}^{n + 1} + A_{W} {\Omega }_{i - 1,j}^{n + 1} + A_{N} {\Omega }_{i,j + 1}^{n + 1} + A_{S} {\Omega }_{i,j - 1}^{n + 1} + S_{p}$$

Here, the following algorithm is used to implement the obtained discretized equations:

- Select a suitable grid. It is recommended to start with $31 \times 31$.
- All dependent variables are initialized to zero.
- The new boundary conditions at the first iteration are calculated
- The new temperature at the current iteration is calculated from previous values at all internal grid points.
- The velocities ($U,V$ and $\theta$) as well as the stream function are calculated in the same way as in step (d).
- The same procedure is followed by starting with step (c)to obtain the solution at the next iteration.
- The iteration process is terminated if the following condition satisfies:
  $$\sum \left| {{\Omega }_{i,j}^{New } - {\Omega }_{i,j}^{old } } \right| \le 10^{ - 6}$$
- The Nusselt and entropy generation are then calculated.

**Figure 2:**
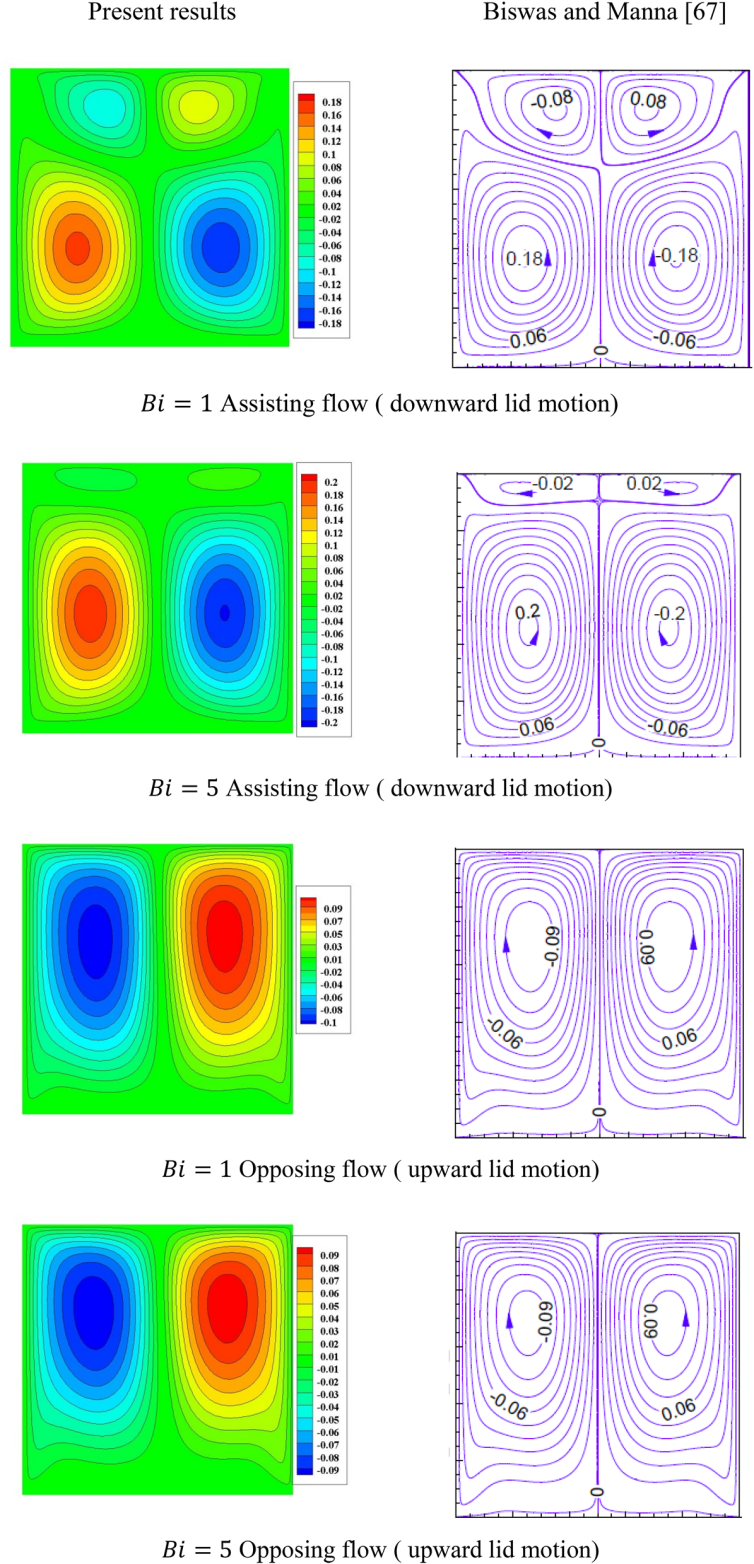
comparison of the streamlines for the different values of Biot number at ${\text{Re}} = 200,\quad {\text{Gr}} = 10^{5} ,\quad {\text{Ma}} = 1000$.

The alternating direction implicit (ADI) is applied to solve the resulting system while the time step is selected to be $10^{ - 4}$. A grid independency investigation is performed and presented in Table 3. It is noted that the grid size of ($121 \times 121)$ is suitable for all the computations. Additionally, there are many validation tests are carried out for the obtained results. Table 4 shows comparisons of the average Nusselt number (at $\beta = 1$) with those obtained by Biswas and Manna^67^. Also, Fig. 2 shows graphical comparisons with Biswas and Manna^67^. All these validation tests show that there are excellent agreements between the outcomes.

**Table 3:** Grid independency study at ${\text{Ra}} = 10^{5} ,\quad {\text{Da}} = 10^{ - 3} ,\quad b = 0.6,\quad d = 0.5,\quad {\text{Ma}} = 1000,\quad \phi = 2\% ,\quad Rd = 1,\quad\Theta = 10^{ - 4} ,\quad N = 0.91,\quad \gamma = 90,\quad \beta = 0.95$.

Grid	$41 \times 41$	$61 \times 61$	$81 \times 81$	$101 \times 101$	$121 \times 121$	$141 \times 141$
${\text{Nu}}_{av}$	5.886469	5.929441	5.954048	5.974059	5.994110	6.015994
$\theta_{cup}$	0.337602	0.341369	0.343260	0.344345	0.344842	0.344917
$\theta_{av}$	0.335150	0.339948	0.341959	0.342499	0.342121	0.340911

**Table 4:** Comparison of the average Nusselt number for the different values of Biot number at ${\text{Re}} = 200,\quad {\text{Gr}} = 10^{5} ,\quad {\text{Ma}} = 1000$.

Bi	Assisting flow ( downward lid motion)		% Errors	Opposing flow ( upward lid motion)		% Errors
	Present results	Biswas and Manna^67^		Present results	Biswas and Manna^67^	
0	10.706	10.729	0.214	10.451	10.364	− 0.839
1	10.736	10.758	0.204	10.418	10.353	− 0.628
5	10.791	10.767	− 0.223	10.383	10.342	− 0.396

## Results and discussion

**Figure 3:**
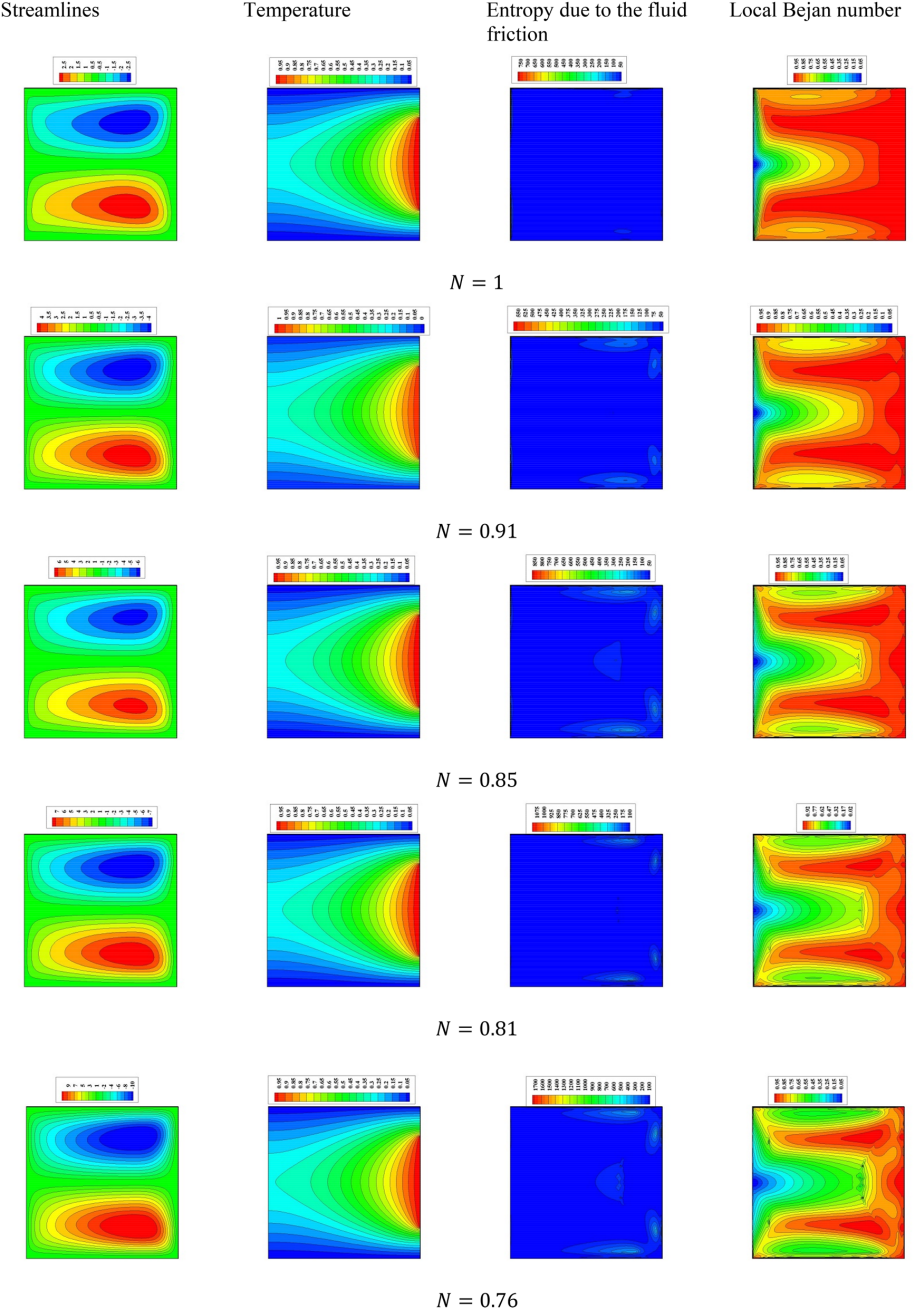
Impacts of the power-law index N on the fluid flow, temperature distributions and entropy generation at ${\text{Ra}} = 10^{5} ,\quad {\text{Da}} = 10^{ - 3} ,\quad b = 0.6, \quad d = 0.5,\quad {\text{Ma}} = 1000,\quad \beta = 0.95,\quad \tau = 1,\quad \phi = 2\% ,\quad Rd = 1,\quad\Theta = 10^{ - 4} ,\quad \gamma = 90$.

**Figure 4:**
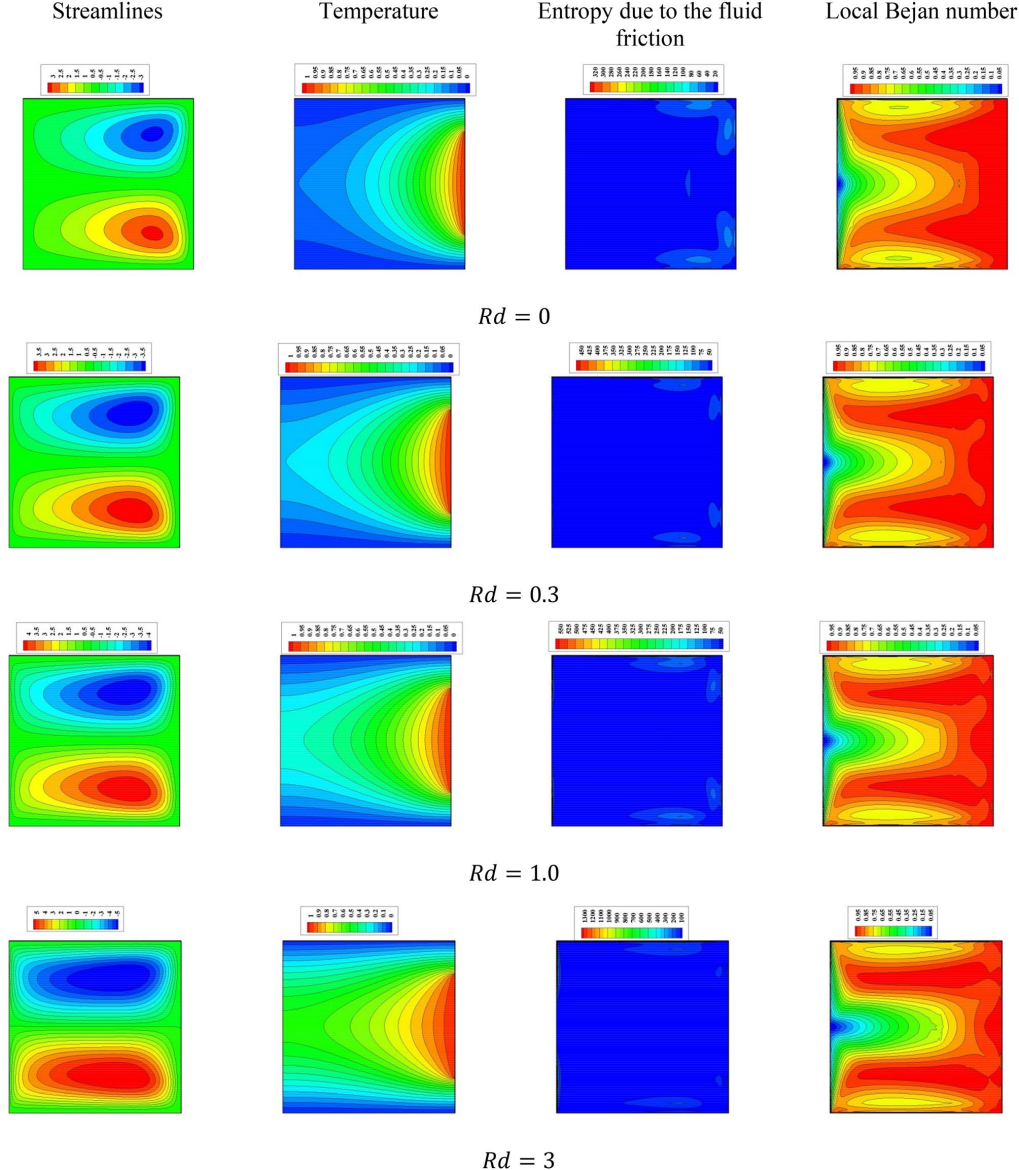
Impacts of the radiation parameter $R$ on the fluid flow, temperature distributions and entropy generation at ${\text{Ra}} = 10^{5} ,\quad {\text{Da}} = 10^{ - 3} ,\quad b = 0.6, \quad d = 0.5,\quad {\text{Ma}} = 1000,\quad \beta = 0.95,\quad \tau = 1,\quad \phi = 2\% ,\quad N = 0.91,\quad\Theta = 10^{ - 4} ,\quad \gamma = 90$.

**Figure 5:**
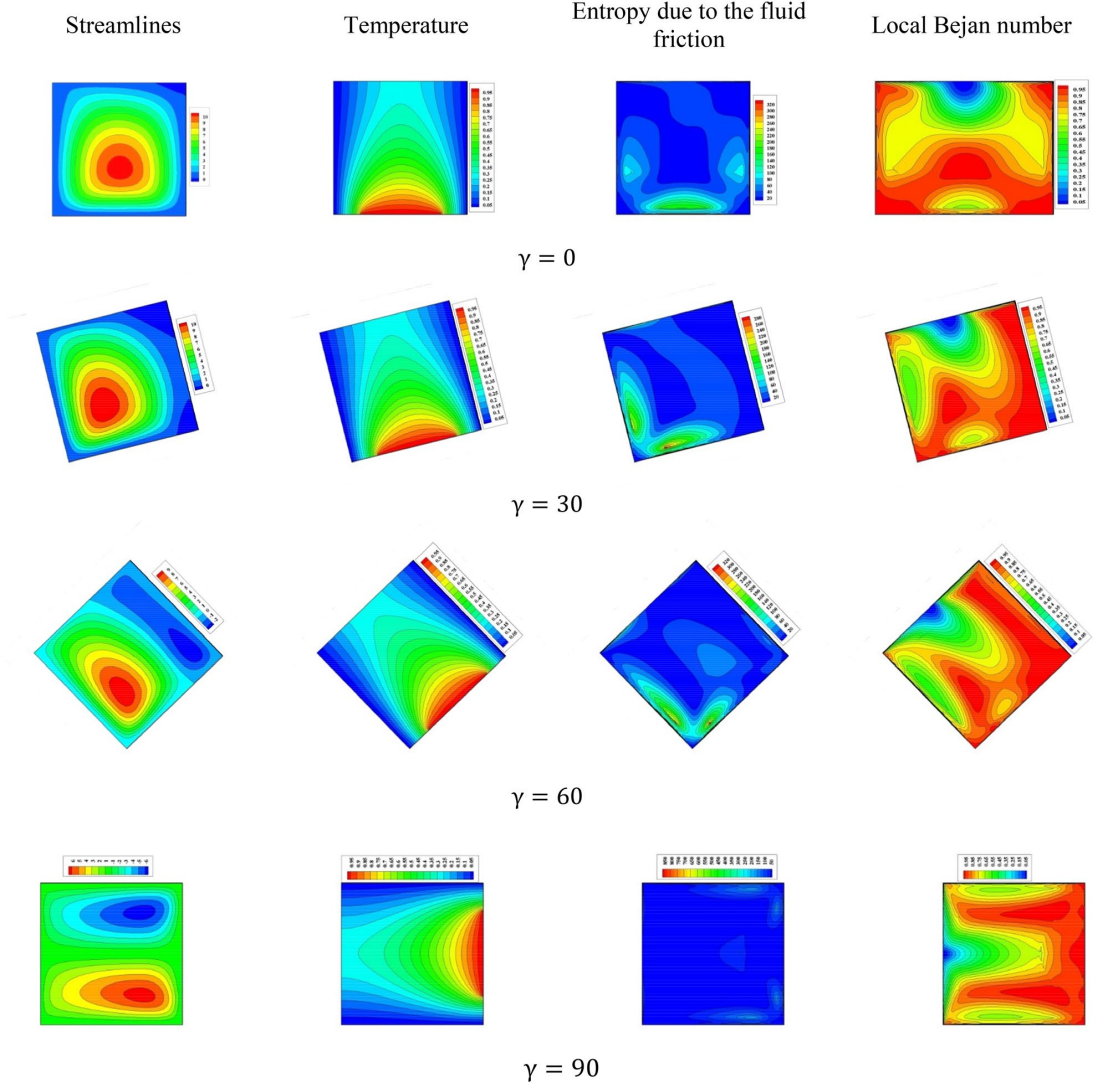
Impacts of the inclination angle $\gamma$ on the fluid flow, temperature distributions and entropy generation at ${\text{Ra}} = 10^{5} ,\quad {\text{Da}} = 10^{ - 3} ,\quad b = 0.6,\quad d = 0.5,\quad {\text{Ma}} = 1000,\quad \beta = 0.95,\quad \tau = 1,\quad \phi = 2\% ,\quad N = 0.85,\quad Rd = 1,\quad\Theta = 10^{ - 4}$.

**Figure 6:**
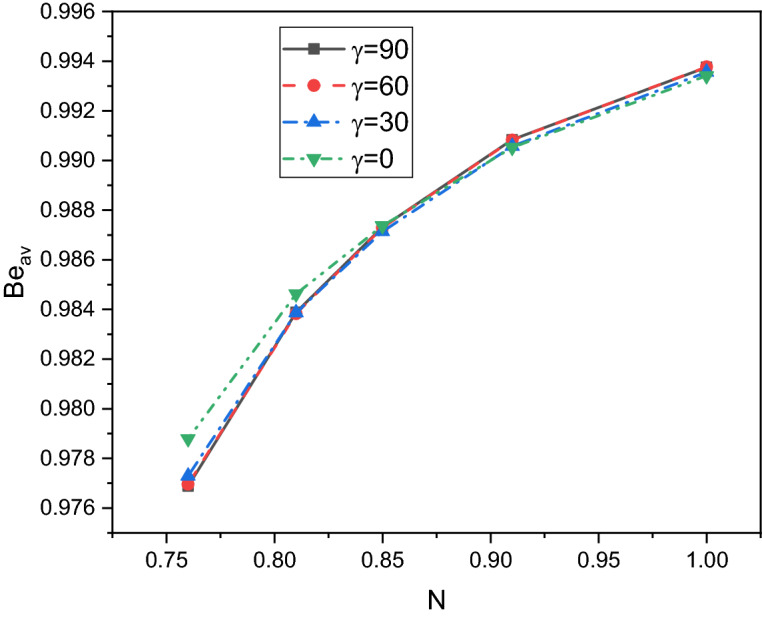
Impacts of the power-law index N and inclination angle $\gamma$ on the average Bejan number at ${\text{Ra}} = 10^{5} ,\quad {\text{Da}} = 10^{ - 3} ,\quad b = 0.6, \quad d = 0.5,\quad {\text{Ma}} = 1000,\quad \beta = 0.95,\quad \tau = 1,\quad \phi = 2\% ,\quad Rd = 1,\quad\Theta = 10^{ - 4}$.

**Figure 7:**
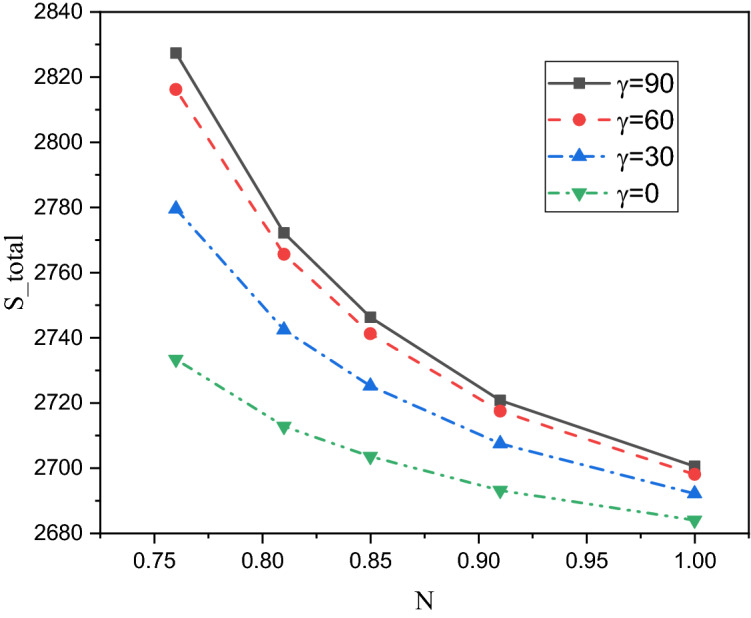
Impacts of the power-law index N and inclination angle $\gamma$ on the total entropy generation at ${\text{Ra}} = 10^{5} ,\quad {\text{Da}} = 10^{ - 3} ,\quad b = 0.6,\quad d = 0.5,\quad {\text{Ma}} = 1000,\quad \beta = 0.95,\quad \tau = 1,\quad \phi = 2\% ,\quad Rd = 1,\quad\Theta = 10^{ - 4}$.

**Figure 8:**
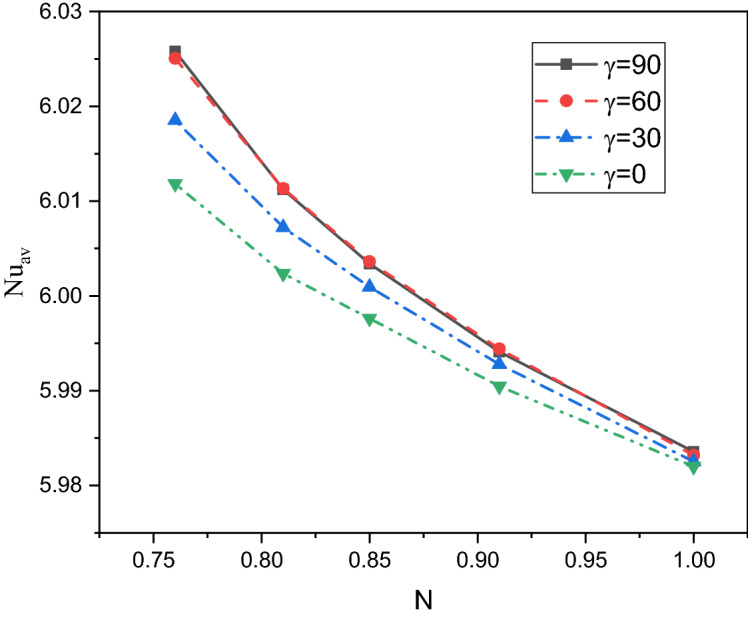
Impacts of the power-law index N and inclination angle $\gamma$ on the average Nusselt number at ${\text{Ra}} = 10^{5} ,\quad {\text{Da}} = 10^{ - 3} ,\quad b = 0.6,\quad d = 0.5,\quad {\text{Ma}} = 1000,\quad \beta = 0.95,\quad \tau = 1,\quad \phi = 2\% ,\quad Rd = 1,\quad\Theta = 10^{ - 4}$.

**Figure 9:**
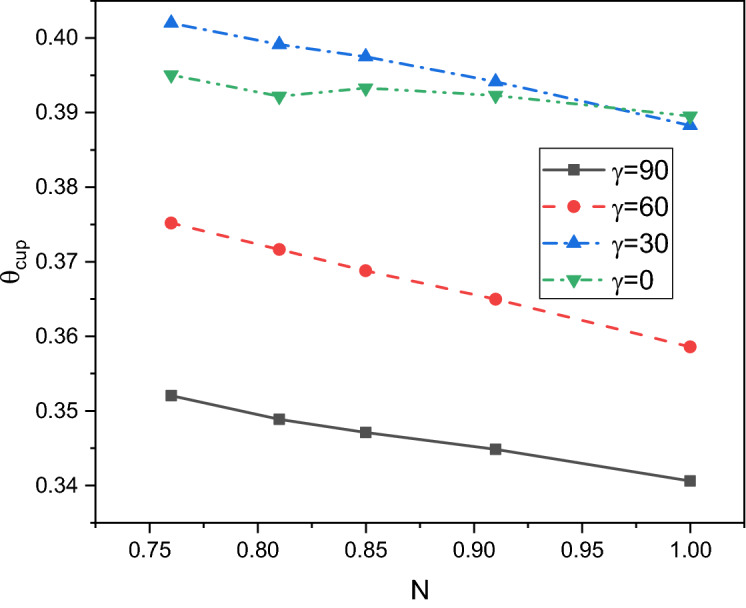
Impacts of the power-law index N and inclination angle $\gamma$ on the cup-mixing temperature at ${\text{Ra}} = 10^{5} ,\quad {\text{Da}} = 10^{ - 3} ,\quad b = 0.6,\quad d = 0.5,\quad {\text{Ma}} = 1000,\quad \beta = 0.95,\quad \tau = 1,\quad \phi = 2\% , \quad Rd = 1,\quad\Theta = 10^{ - 4}$.

**Figure 10:**
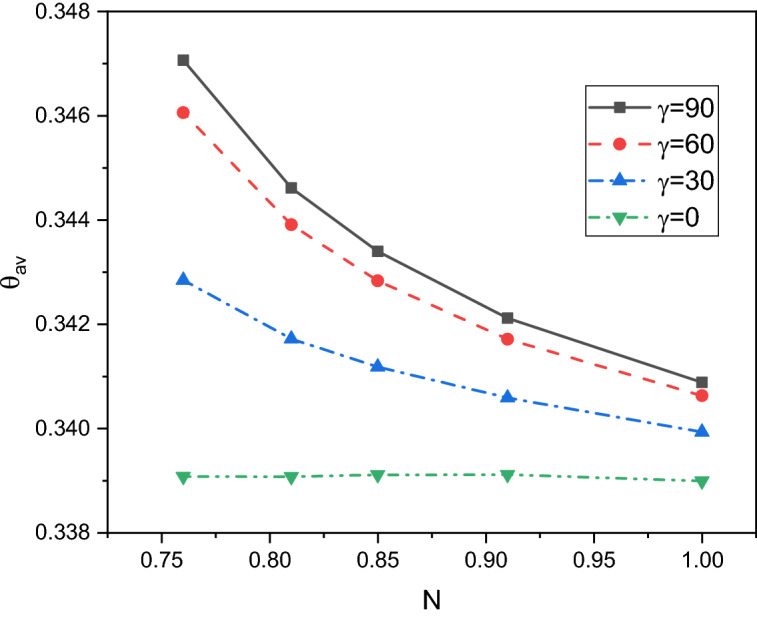
Impacts of the power-law index N and inclination angle $\gamma$ on the bulk-averaged temperature at ${\text{Ra}} = 10^{5} ,\quad {\text{Da}} = 10^{ - 3} ,\quad b = 0.6,\quad d = 0.5,\quad {\text{Ma}} = 1000,\quad \beta = 0.95,\quad \tau = 1,\quad \phi = 2\% , \quad Rd = 1,\quad\Theta = 10^{ - 4}$.

A comprehensive discussion of the obtained outcomes is presented in this section. Here, it is interested with the influences of the time parameter ($0.1 \le \tau \le 0.5)$ (unsteady state), variations of the fractional order ($0.8 \le \beta \le 1)$, the power-law index ($0.76 \le N \le 1)$, the radiation parameter ($0 \le Rd \le 3)$ and the inclination angle ($0 \le \gamma \le \pi /2)$. Also, the corresponding value of the Prandtl number is set as ${\text{Pr}} = 204$. The outcomes presentation tools are the contours of the streamlines, isotherms, entropy due to fluid friction and local Bejan number. Also, graphical illustrations for the average Nusselt number, cup-mixing temperature, bulk-averaged temperature, total entropy and average Bejan number are taken into account. Impacts of the fraction derivatives order $\beta$ and dimensionless time parameter $\tau$ on the maximum values of the stream function, cup-mixing temperature, bulk-averaged temperature and average Nusselt number at ${\text{Ra}} = 10^{5} ,\quad {\text{Da}} = 10^{ - 3} ,\quad b = 0.6,\quad d = 0.5, \quad {\text{Ma}} = 1000,\quad \phi = 2\% ,\quad Rd = 1,\quad\Theta = 10^{ - 4} ,\quad N = 0.91, \quad \gamma = 90$ are presented in Table 5. Here, effects of the fraction order are examined for various cases of the unsteady flows. It is noted that as the fractional order approaches to one, a clear reduction in values of the maximum stream function, cup-mixing temperature and bulk-averaged temperature is obtained. On the contrary, values of the average Nusselt number are enhanced with the increase in the fractional order. These behaviors are noted for all the considered values of the time. In the same context, as the time is progressed, the maximum values of the stream function, cup-mixing temperature and bulk-averaged temperature are enhanced while the average Nusselt number is reduced. Features of the streamlines, temperature, irreversibility of the fluid friction and local Bejan number for the alteration of the power-law index $N$ are shown in Fig. 3. These findings are conducted at ${\text{Ra}} = 10^{5} ,\quad {\text{Da}} = 10^{ - 3} ,\quad b = 0.6,\quad d = 0.5,\quad {\text{Ma}} = 1000,\quad \beta = 0.95,\quad \tau = 1,\quad \phi = 2\% ,\quad Rd = 1,\quad\Theta = 10^{ - 4} ,\quad \gamma = 90$. It is noticeable that the increase in the power-law index causes a weakness in the mixture flow while the temperature distributions are not much affected by the variations of $N$. Physically, the increase in $N$ results in a supporting in the overall dynamic viscosity and hence the nanofluid flow is slowdown. Like effects of $N$ on the streamlines, the irreversibility due to the fluid friction is diminished as $N$ approaches to one. This behavior returns to the decrease in the velocity gradients due to the enhancement in the dynamic viscosity. Additionally, features of the local Bejan number show a dominance of the heat transfer entropy at high values of $N$ comparing with the fluid friction entropy due to the increase in the thermal boundary layers. Impacts of the radiation parameter $Rd$ on the streamlines, temperature, entropy due to the fluid friction and local Bejan number at ${\text{Ra}} = 10^{5} ,\quad {\text{Da}} = 10^{ - 3} , \quad b = 0.6,\quad d = 0.5,\quad {\text{Ma}} = 1000,\quad \beta = 0.95,\quad \tau = 1,\quad \phi = 2\% , \quad N = 0.91,\quad { }\Theta = 10^{ - 4} ,\quad \gamma = 90$ are examined using Fig. 4. The results indicated that significant augmentations in both the mixture flow and thermal boundary layers are given as $Rd$ is altered. The physical explanations of these observations are due the extra heat flux obtained from the presence of the radiation that causes an increase in the buoyancy force. Also, the gradients of the velocity are enhanced as $Rd$ is increased which causes a supporting in both values and distributions of the fluid friction entropy. In the same context, features of the local Bejan number show that the dominance of the heat transfer irreversibility is decreased as $Rd$ is increased due to the increase of dominance of the fluid friction irreversibility on the flow domain. In Fig. 5, various configurations of the flow features are noted as the inclination angle is altered. At this point, the flow features are represented by a major anti-clockwise circular vortex at the low values of $\gamma$ ($\gamma = 0, 30$). However, as $\gamma$ is varied ($\gamma = 60)$, a minor clockwise vortex is formulated near the right wall. This cell is enlarged as $\gamma$ is increased until a symmetrically flow is obtained at $\gamma = 90$. The temperature distributions show an enhancement in the temperature gradients as $\gamma$ is increased indicating a good rate of the heat transfer at $\gamma = 90$. The fluid friction entropy indicates that the fluid friction irreversibility is occurred near the left and bottom walls while as $\gamma$ is increased, values of the fluid friction entropy is enhanced due to the enhancement of the velocity gradients. Figure 6 displays the profiles of the average Bejan number for the variations of the inclination angle $\gamma$ and the power-law index $N$. It is noted that ${\text{Be}}_{av} > 0.5$ for all values of $\gamma$ and $N$ which indicating to the dominance of the heat transfer entropy comparing with the fluid friction entropy. The results, also, disclosed that the increase in the power-law index enhances the temperature gradients and hence the average Bejan number is augmented. Figure 7 exhibits that the total entropy confined the flow domain is a decreasing function in the power-law index $N$ due to the increase in the dynamic viscosity while as the inclination angle $\gamma$ is growing, an enhancement in the temperature differences are obtained and hence $S_{total}$ is supported. Impacts of $\gamma$ and $N$ on values of the average Nusselt number ${\text{Nu}}_{av}$ are examined with the help of Fig. 8. The figure revealed that the growing in the power-law index $N$ causes a reduction in the rate of the heat transfer while the thermal boundary layer near the heated section is enhanced as $\gamma$ approaches to 90. The cup-mixing temperature shows the inverse behavior of the average Nusselt number when the impacts of $\gamma$ is examined. These observations are presented in Fig. 9. It is, also, noted that the power-law index $N$ has a negative effects on the cup-mixing temperature. In the same context, Fig. 10 presents the profiles of the bulk-averaged temperature $\theta_{av}$ for the different values of $\gamma$ and $N$ at ${\text{Ra}} = 10^{5} ,\quad {\text{Da}} = 10^{ - 3} ,\quad b = 0.6,\quad d = 0.5,\quad {\text{Ma}} = 1000,\quad \beta = 0.95,\quad \tau = 1,\quad \phi = 2\% ,\quad Rd = 1,\quad\Theta = 10^{ - 4}$. It is remarkable that the changing in values of $\gamma$ enhances the bulk-averaged temperature while the opposite observations are found when the power-law index $N$ is growing. All these behaviors are due the increase in the overall dynamic viscosity that reduces the convective-radiation mode.

**Table 5:** Impacts of the fraction derivatives order $\beta$ and dimensionless time parameter $\tau$ on the maximum values of the stream function, cup-mixing temperature, bulk-averaged temperature and average Nusselt number at ${\text{Ra}} = 10^{5} ,\quad {\text{Da}} = 10^{ - 3} ,\quad b = 0.6,\quad d = 0.5,\quad {\text{Ma}} = 1000,\quad \phi = 2\% ,\quad Rd = 1,\quad\Theta = 10^{ - 4} ,\quad N = 0.91,\quad \gamma = 90$.

$\tau$	$\beta$	$\psi_{max}$	$\theta_{cup}$	$\theta_{av}$	${\text{Nu}}_{av}$
0.1	0.8	1.909413	0.324477	0.250873	6.904846
	0.85	1.909034	0.324467	0.250828	6.905488
	0.9	1.908571	0.324457	0.250773	6.906251
	0.95	1.908004	0.324442	0.250710	6.907175
	1.0	1.907308	0.324425	0.250632	6.908265
0.2	0.8	3.339026	0.334704	0.305505	6.305857
	0.85	3.338609	0.334694	0.305474	6.306139
	0.90	3.338117	0.334685	0.305438	6.306466
	0.95	3.337536	0.334674	0.305397	6.306841
	1.0	3.336846	0.334662	0.305350	6.307249
0.5	0.8	4.536020	0.344846	0.342137	5.993988
	0.85	4.535823	0.344844	0.342133	5.994018
	0.9	4.535704	0.344843	0.342127	5.994056
	0.95	4.535573	0.344842	0.342121	5.994108
	1.0	4.535487	0.344841	0.342115	5.994159

## Conclusions

Using the fractional derivatives basics, the unsteady convective-radiation flow confined an enclosure filled with CMC-water power-law non-Newtonian nanofluids was investigated. The fractional derivatives were taken on the time while the conformable definitions were used to approximate the calculations. The Marangoni effects are imposed to the top-free surface of the domain while the bottom boundaries are partially heated. The one-phase model in which the overall dynamic viscosity and thermal conductivity are functions of the power-law index is presented while the Rosseland approximation is used for the thermal radiation. Beside the cup-mixing temperature and the bulk-averaged temperature, the entropy of the system is examined for the variations of the controlling parameter. The main outcomes of this study revealed that the increase in the fractional order enhances the average Nusselt number while the maximum values of the stream function, the cup-mixing temperature and the bulk-averaged temperature are reduced as $\beta$ approaches to one, regardless values of the time. Also, presence of the radiation parameter within the domain accelerates the mixture flow and enhances the thermal boundary layer. Additionally, the increase in the power-law index reduces the convective mode, the total entropy, the cup-mixing temperature and the bulk-averaged temperature while the average Nusselt number is enhanced.

## List of symbols

$c_{p}$: Specific heat at constant pressure
Da: Darcy Number
$${\text{g}}$$: Gravity acceleration
$$H$$: Length
$$K$$: Permeability
$$k$$: Thermal conductivity
Ma: Marangoni number
$N$: Power-law index
Nu: Local Nusselt number
${\text{Nu}}_{av}$: Average Nusselt number
$$P$$: Pressure
Pr: Prandtl number
Ra: Rayleigh number
$$Rd$$: Radiation parameter
$$T$$: Temperature
$$t$$: Time
$\left( {u,v} \right)$: Velocity components in the *x* and *y* direction
$\left( {U,V} \right)$: Dimensionless velocity components in the *x* and *y* direction
$\left( {x,y} \right)$: Dimensional Cartesian coordinates
$\left( {X,Y} \right)$: Dimensionless Cartesian coordinates

## Greek symbols

$$\alpha_{f}$$: Thermal diffusivity
$\beta$: Coefficient of thermal expansion
$$\upgamma$$: Inclination angle
$\upvarepsilon$: Porosity of the porous media
$$\uptheta$$: Dimensionless temperature
$$\upmu$$: Dynamic viscosity
$$\upnu$$: Kinematic viscosity
$$\uprho$$: Density
$$\upsigma$$: Surface tension
$\tau_{ij}$: Shear stress tensor
τ: Dimensionless time
$\phi$: Solid volume fraction

## Subscripts

$$eff$$: Effective
$$f$$: Fluid
$$nf$$: Nanofluid
$$p$$: Porous media
$$c$$: Cold
$$h$$: Hot
